# *Cytauxzoon* sp. Infection and Coinfections in Three Domestic Cats in Central Italy

**DOI:** 10.3390/vetsci9020050

**Published:** 2022-01-27

**Authors:** Maria Teresa Antognoni, Francesca Rocconi, Silvia Ravagnan, Marta Vascellari, Gioia Capelli, Arianna Miglio, Morena Di Tommaso

**Affiliations:** 1 Department of Veterinary Medicine, University of Perugia, Via San Costanzo 4, 06126 Perugia, PG, Italy; maria.antognoni@unipg.it; 2 Faculty of Veterinary Medicine, Veterinary University Hospital, University of Teramo, Località Piano D’Accio, 64100 Teramo, TE, Italy; frocconi@unite.it; 3 Istituto Zooprofilattico Sperimentale delle Venezie, Viale dell’Università 10, 35020 Legnaro, PD, Italy; sravagnan@izsvenezie.it (S.R.); mvascellari@izsvenezie.it (M.V.); gcapelli@izsvenezie.it (G.C.)

**Keywords:** *Cytauxzoon* sp., *Candidatus* Mycoplasma turicensis, coinfection, cat, Italy

## Abstract

Cytauxzoonosis is an emerging disease caused by a tick-transmitted haemoprotozoan affecting domestic and wild felids. The clinical and biomolecular findings of the infection due to *Cytauxzoon* sp. and concomitant coinfections are described in three cats in central Italy. Three domestic cats were referred for different clinical conditions (impact trauma, lameness, and weight loss and lethargy). They presented different hematobiochemical profiles. Only two cats were anemic, but in all three cats, endo erythrocyte inclusions suggestive of piroplasmids were found at blood smear evaluation. EDTA blood samples were submitted to rapid ELISA test for feline immunodeficiency virus (FIV) and feline leukemia virus (FeLV), and to biomolecular investigations for Piroplasmida (*Babesia* spp., *Theileria* spp., *Cytauxzoon* spp.) and *Mycoplasma* spp. All three cats were positive for *Cytauxzoon* sp. (European *Cytauxzoon* species) and two cases were also coinfected by *Candidatus* Mycoplasma turicensis and FIV. This report suggests that cytauxzoonosis should be included among differential diagnoses in subjects with possibility of contact with ticks and with presence of coinfections by tick-borne parasites, including in non-endemic areas.

## 1. Introduction

Cytauxzoonosis is an emerging tick-borne disease that affects domestic cats and wild felids and is caused by apicomplexan protozoan haemoparasites (family Theileriidae) belonging to the genus *Cytauxzoon*. Organisms of the genus *Cytauxzoon* exist in two distinct tissue forms: an erythrocyte phase, termed piroplasm, and a tissue phase, named schizont [1]. Several species have been identified. *Cytauxzoon felis* represents the main species with different genotypes [2,3]. For many years, *Cytauxzoon felis* was reported only in North America, particularly in the mid-Atlantic states of the United States [4,5,6,7,8,9,10,11]. *Dermacentor variabilis* and *Amblyomma americanum* have been shown to be the tick vectors of *Cytauxzoon felis* [12,13,14,15,16,17,18,19]. Bobcats (*Lynx rufus*) have been considered as the natural reservoir of *Cytauxzoon felis* and show up to 100% prevalence (PCR-positive and parasitemic) in some enzootic areas (e.g., mid-Atlantic states of the United States) [2,20,21]. In North America, *Cytauxzoon felis* has also been identified in other wild felids, such as pumas (*Puma concolor*) and panthers (*Puma concolor coryi*) [22,23,24]. In the early 2000s, *Cytauxzoon felis* was reported in Brazil and in other geographic areas (e.g., South Africa, China, Iran, Turkey, Spain), both in wild felids, such as jaguars (*Panthera onca*), lion (*Panthera leo*), ocelots (*Leopardus pardalis*), cheetahs (*Acinonyx jubatus*), little spotted cat (*Leopardus tigrinus*), Iberian lynx *(Lynx pardinus)*, wild cat (*Felis silvestris*) and the domestic cat [25,26,27,28,29,30,31,32,33,34,35,36,37].

Phylogenetically, *Cytauxzoon felis* isolates from USA and Brazil belong to the same subclasses, different from Mongolian *Cytauxzoon manul* and European isolates from the Old World, European *Cytauxzoon* species [38]. In particular, *Cytauxzoon manul* was reported in the free-ranging Pallas cat (*Otocolobus manul*) in Mongolia and in the lion (Zimbabwe), while *Cytauxzoon* sp. has been reported in Europe and in other geographic areas (e.g., South Africa, India, Turkey, Japan, Brazil), both in domestic cats and in wild felids [32,39,40,41,42]. The only species reported to be infected by *Cytauxzoon* sp. not belonging to the Felidae family is the meerkat (*Suricata suricatta*) in South Africa and the Hokkaido brown bear in Japan [43,44].

To our knowledge, European reports of *Cytauxzoon* sp. infection concern both domestic cats from France, Italy, Switzerland, Spain, Germany, Portugal, and the European wild cat (*Felis silvestris silvestris*), lynx (*Lynx pardinus*, *Lynx lynx*) both considered reservoirs and incidental hosts [45,46,47,48,49,50,51,52]. Epidemiological studies on *Cytauxzoon* species in Europe showed different prevalence, ranging from 0% in Greece, 0.8% in France to 23% in Italy, although data varies according to geographic area [53,54,55,56]. Data regarding the presence and distribution of *Cytauxzoon* sp. in Italy are few and limited to single areas, mainly involving northern and central Italy, with conflicting results on the prevalence of the pathogen [55,57,58,59,60,61]. Moreover, there is only one report describing clinical cases of *Cytauxzoon* sp. in two free-ranging cats [49]. It is hypothesized that ticks, such as *Ixodes ricinus* and *Dermacentor*, may be involved in the transmission of *Cytauxzoon* sp. infection, but the vector remains unknown as well as other possible modes of transmission [12,55,62]. The arthropods collected from positive cats from north-eastern Italy were all negative using PCR for *Cytauxzoon* sp. [49].

Based on clinical features, for many years, *Cytauxzoon felis* in domestic cats (*Felis catus*) without treatment was reported to be associated with the development of acute and often fatal disease (97% mortality), characterized by clinical signs, such as anemia, icterus, depression, lethargy, dyspnea, tachycardia, vomiting, inappetence, anorexia, splenomegaly, hepatomegaly, fever, generalized pain, and vocalization. Ischemic neuropathological alterations, due to the formation of parasitic emboli, were also documented. A report of a possible association between *Cytauxzoon felis* infection and cardiac trifascicular block in a cat was described [1,7,63,64]. Recent studies have reported that cats surviving *Cytauxzoon felis* infection maintain subclinical and persistent parasitemia and that they represent reservoirs of infection for other felids [2,7,16,65,66,67,68,69]. Possible repeat infection and illness are also described [70]. In endemic areas (e.g., North America) the prevalence of the subclinical form in cats may be as high as 30% [2]. *Cytauxzoon* sp. in Europe seems less virulent than *Cytauxzoon felis* and the clinical manifestations appear only in cases of concurrent diseases or immunodeficiency (e.g., immune-mediated diseases or secondary infections) [46,51,55,56]. Fever, anemia, lethargy, weight loss, vomiting, and diarrhea have occasionally been reported in association with *Cytauxzoon* sp. infection [48,49,55]. To date, epidemiological studies conducted in various countries show the existence of areas considered endemic for the spread of *Cytauxzoon* sp., while there are areas in which its presence has never been reported. Currently, there is no clear picture of the present European spread of this parasite, and the reporting of clinical cases in areas not yet considered enzootic is useful for directing future epidemiological and clinical studies focused on this scarcely described infectious disease.

Therefore, the present study provides a clinical and biomolecular description of infection by *Cytauxzoon* sp. in three domestic cats in central Italy (Umbria region) with the aim of increasing the epidemiological, clinical, and biomolecular data regarding this parasitosis in a non-endemic area. 

## 2. Case Presentations

**Figure 1:**
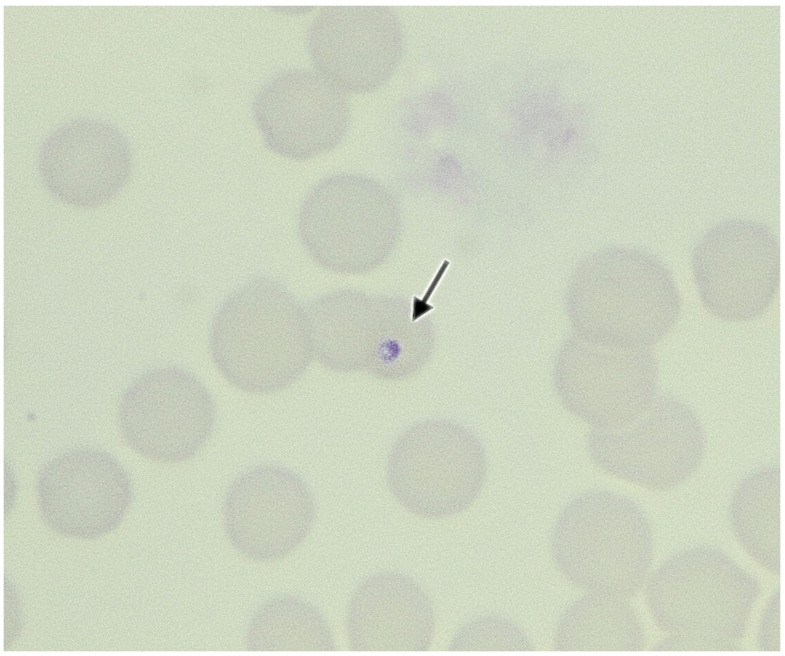
Stained blood smear showing a merozoite (arrow) within an erythrocyte in a domestic cat in central Italy. May–Grunwald–Giemsa stain, magnification of 100×.

**Cat n. 1** was a two-year-old, spayed female, domestic shorthair weighing 3.2 kg that was referred for impact trauma to the Veterinary Teaching University Hospital of Perugia in October 2018. The cat had an outdoor lifestyle, was vaccinated and received irregular ectoparasiticide treatments. The cat presented pale mucous membranes and jaundice, a mild grade of dehydration (3%), and opaque fur. There was also a retraction of the right globe and lateral right mandibular deviation with loss of teeth and pain. She also had ulcers in the oral and nasal mucosa. At clinical presentation, no ticks or fleas were found. Complete blood count (CBC) (Sysmex XT-2000iV; Sysmex, Kobe, Japan) showed moderate normocytic and normocromic anemia and reduction of platelet count with increased mean platelet volume. Blood smear evaluation using the May–Grunwald–Giemsa stain (Aerospray Slide Stainer 7120, Delcon^®^, Bergamo, Italy) enabled identification of macroplatelets and confirmed trombocitopenia and the presence of endo erythrocyte inclusions suggestive of piroplasmids (*Cytauxzoon* spp., *Theileria* spp., *Babesia* spp.) (Figure 1). The parasitemia was classified as mild grade (two erythrocytes parasized hpf) [55]. The biochemical profile (Hitachi 904, Boehringer Mannheim and Seac Radim reagents Biolabo sas, Les Hautes, France) showed hyperbilirubinemia, and mild increase in ALT, ALP, cholesterol, urea, and LDH. A test for feline leukaemia virus (FeLV) and feline immunodeficiency virus (FIV) using a rapid enzyme-linked immunosorbent assay (SNAP Combo Plus FeLV ag/FIV ab test, Idexx Laboratories Westbrook, ME, USA) revealed a negative result for FeLV and a positive result for FIV (Table 1). Since *Cytauxzoon* sp. is morphologically indistinguishable from other piroplasms, such as *Babesia* spp. or *Theileria* spp., and may be mistaken as *Mycoplasma haemofelis* [63], an EDTA blood sample was submitted for biomolecular investigations for these pathogens to obtain a definitive diagnosis. This patient was positive for *Cytauxzoon* sp. and *Candidatus* Mycoplasma turicensis.

The cat was hospitalized and was submitted to oxygen therapy and intravenous crystalloid fluid therapy was given to correct the dehydration. Surgical stabilization of the mandible and placement of nasogastric feeding tube was performed. Antibiotic treatment with doxycycline (10 mg/kg/q12h PO for 21 days, Vibravet, Zoetis, Italy) was started, both as an initial therapy for suspected mycoplasmosis [48,49], and as a preventive treatment for concomitant infections after trauma injury. Since molecular investigations (day 10) showed positivity for mycoplasma, it was decided to continue the administration of doxycycline and to add imidocarb dipropionate for cytauxzoonosis. The cat was discharged after two weeks of hospitalization following normalization of clinical signs. Upon discharge, it was recommended to continue therapy with doxycycline. Unfortunately, there are no data on follow-up.

**Cat n. 2** was a six-year-old, 5.2 kg, neutered male, domestic shorthair referred by a colleague for evaluation of right hind limb lameness in May 2019. The cat had regular outdoor access and had a history of irregular vaccinations and ectoparasiticide treatments. On presentation, the cat showed lameness and the physical examination was otherwise unremarkable. No ticks or fleas were found. Hematological and biochemical analysis were performed, and the results are reported in Table 1. CBC showed mild lymphopenia. Blood smear evaluation revealed the presence of endo erythrocyte inclusions suggestive of piroplasmids. The parasitemia was classified of mild grade (one erythrocyte parasized/hpf). The biochemical profile showed moderate increase in ALT and ALP, and mild increase in urea, glycemia, calcium and phosphorus. Tests for FeLV and FIV were negative. Radiographic examination performed showed a partially consolidated, displaced distal fracture of the right femur. A sample of EDTA blood was submitted for biomolecular investigation for Piroplasmida (*Babesia* spp., *Theileria* spp., *Cytauxzoon* spp.) and *Mycoplasma* spp. which showed positivity for *Cytauxzoon* sp. and *Candidatus* Mycoplasma turicensis. 

The patient was observed for two days, the time necessary to perform collateral laboratory and instrumental investigations. As the fracture was old, surgical intervention was excluded and enforced rest was recommended. The cat was treated with doxycycline (10 mg/kg/q12h PO, Vibravet, Zoetis, Italy). After confirmation by PCR of *Cytauxzoon* sp. infection and sequencing, doxycycline was discontinued and imidocarb dipropionate was administered (5 mg/kg IM twice, two weeks apart, Carbesia, Intervet, Italy) at days 7 and 21 with improvement of clinical conditions. No follow up data are available.

**Cat n. 3** was an 8-year-old, neutered female, 2.5 kg, indoor/outdoor, domestic shorthair. The cat had a history of tick exposure and irregular preventative ectoparasiticide treatments. The patient presented weight loss, depression, dysorexia, lethargy, emaciation (body condition score 3/9), moderate dehydration (5%), sialorrhea, and submandibular lymphadenomegaly. Oral examination showed a nodule. The hematobiochemical profile is summarized in Table 1. CBC showed severe microcytic normocromic anemia, lymphocytosis and decreased platelet count. At blood smear evaluation, endo erythrocyte inclusions suggestive of piroplasmids were found and the parasitemia was classified as mild grade (one erythrocyte parasized/hpf). The biochemical profile revealed moderately increased AST, urea, LDH and moderate hypoalbuminemia. The cat tested negative for FeLV and positive for FIV. The lesion, submitted to fine needle aspiration cytological assessment, was diagnosed as an epithelial neoplasm (suspected salivary gland adenocarcinoma). A sample of EDTA blood was submitted for biomolecular investigation for Piroplasmida (*Babesia* spp., *Theileria* spp., *Cytauxzoon* spp.) and *Mycoplasma* spp. showing a positive result for *Cytauxzoon* sp. 

The cat was hospitalized and fluid therapy with crystalloid solutions was administered. The owners rejected surgical treatment for mass excision. Therefore, only medical treatment with doxycycline (10 mg/kg, orally once a day, Vibravet, Zoetis, Italy) was performed. Before receiving the results of the biomolecular investigation, the general condition of the cat worsened, and the owners agreed to euthanasia, but necropsy was denied. 

## 3. Biomolecular Analysis

The DNA was extracted from EDTA blood using the High Pure PCR Template Preparation kit (Roche Diagnostics, Munich, Germany) according to the manufacturer’s instructions. Piroplasmida (*Babesia* spp., *Theileria* spp., *Cytauxzoon* spp.) detection was performed using an SYBR Green real-time PCR with the primers BJ1-BN2 previously described [71]. The reactions were carried out in a total volume of 20 µL, containing 10 µL of QuantiFast SYBR Green PCR Master mix 2X (Qiagen GmbH, Germany), 0.1 µM of sense and reverse primers and 3 µL of extracted DNA. Amplifications were performed in a StepOnePlus™ instrument (Applied Biosystems, Foster City, CA, USA). The thermal profile consisted of 5 min of activation at 95 °C, followed by 40 cycles at 95 °C for 15 s and 60 °C for 1 min. Following amplification, a melting curve analysis was performed by slowly raising the temperature of the thermal chamber from 60 °C to 95 °C to discriminate between specific amplicons and non-specific amplification products. The samples were also screened for *Mycoplasma* spp. using the primers from Willy et al., 2009 [72]. The *Mycoplasma* spp. positive samples were amplified with the primers MycE929f- MycE1182r (16S rRNA gene) to allow the sequencing and identification of the species [73].

Amplification products were directly sequenced to identify the species. All PCR products were purified and sequenced in both directions using the same forward and reverse primers using the ABI PRISM 3130 Genetic Analyzer (Applied Biosystems, Carlsbad, CA, USA). Nucleotide sequences were compared with representative sequences available in GenBank using the Basic Local Alignment Search Tool (BLAST). Phylogenetic analyses were carried out using the neighbor-joining (N-J) method, with 1000 bootstrap replicates implemented in the MEGA 6 program [74].

The three samples tested positive to Piroplasmida PCR assay, and the sequences showed positivity to *Cytauxzoon* sp. Cat n. 1 and cat n. 2 results indicated co-infection with *Candidatus* Mycoplasma turicensis.

The sequences of cat n. 1 and cat n. 3 presented 100% query cover and 100% identity with *Cytauxzoon* sp. sequences isolated from cats from Germany and Switzerland (MN629916, MF503146). The sequence of cat n. 2 presented one nucleotide of difference with respect to the first two and presented 100% query cover and 100% identity with *Cytauxzoon* sp. sequences isolated from cats from Switzerland, Romania and France and from the Iberian lynx of Spain (MF503148, KT361081, EU622908 and EF094470). The sequences were deposited in GenBank under the accession numbers OM281707 (cat n.1), OM281708 (cat n.2) and OM281709 (cat n.3).

**Figure 2:**
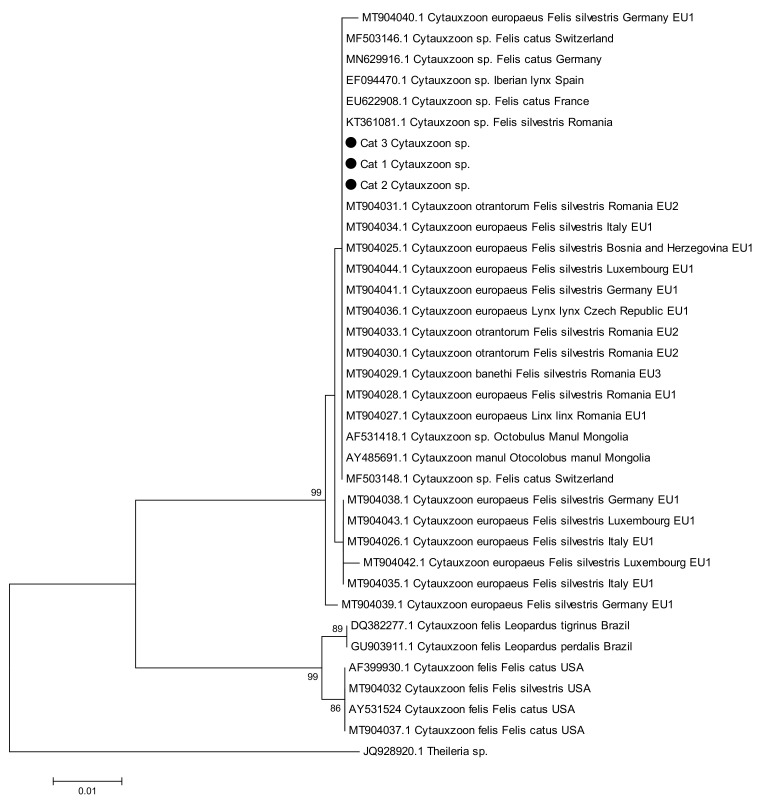
Phylogenetic tree based on 18S rDNA gene partial sequences (~445 bp) of *Cytauxzoon* spp. (all *Cytauxzoon* species). The sequence dataset was analyzed using MEGA v6.0. The tree was constructed using the neighbor-joining (NJ) method and bootstrap analysis (1000 replicates) based on the ClustalW algorithm. Significant bootstrapping values (>70%) are shown on the nodes. *Theileria* spp. was used as outgroup. The sequences of this study, accession numbers OM281707–OM281709, are indicated by black dot (●).

The phylogenetic analysis, based on an alignment of the sequences of this study and representative *Cytauxzoon* spp. sequences of partial 18S rDNA (Figure 2), showed that our isolates clustered with European *Cytauxzoon* sequences, which include the genotypes *Cytauxzoon europeaus* (EU1 genotype)*, Cytauxzoon otrantorum* (EU2 genotype), and *Cytauxzoon benethi* (EU3 genotype) [38], and *Cytauxzoon manul*. *Cytauxzoon felis* from Brazil and USA belonged to a different cluster.

## 4. Discussion

The present study describes *Cytauxzoon* sp. infection in three autochthone cats living in central Italy (Umbria region), considered a non-endemic area. Furthermore, we describe, for the first time, the coinfection of this parasite with *Candidatus* Mycoplasma turicensis and FIV in domestic cats.

In the present study, all the cats were undergoing other diagnostic procedures. The detection of parasites in red blood cells at microscopic examination was accidental and the infection was confirmed by molecular analysis. In particular, cat n. 1 presented pale and subicteric mucous membranes and ulcers in the oral and nasal mucosa. Since these symptoms were non-specific and common to other coexisting diseases in the subject, it was not possible to determine with certainty the correlation with *Cytauxzoon* sp. Cat n. 2 showed no symptoms other than lameness, whereas cat n. 3 showed clinical signs including weight loss, depression, dysorexia, lethargy, emaciation, and sialorrhea, probably associated with oral neoplasia rather than parasite infection. 

Clinical data on *Cytauxzoon* sp. infection in the domestic cat in Europe have been sporadically reported. For example, case reports have been produced in France, Italy, Switzerland, Germany and Portugal [46,48,49,50,51,55]. Previous cases reported have reported fatal outcomes, but the majority of infected cats were apparently healthy [48,50,51,55,56]. The clinical signs reported in previous studies included hyperthermia (>40 °C), gastrointestinal signs (e.g., diarrhea, vomiting), weight loss, anorexia, abdominal pain, stomatitis, ulcerative dermatitis, lethargy, circling, vocalizations, icterus, and anemia [7]. Pancytopenia, splenomegaly and hepatomegaly are also common [75].

All the subjects in the present study had a low level of erythroparasitaemia. This data was in accordance with the study by Carli et al., which showed subclinical erythroparasitaemia in the majority of infected cats [55]. Regarding *Cytauxzoon felis*, the erythrocytic (piroplasmatic) phase is relatively innocuous and does not cause severe clinical signs in cats, whereas the tissue (schizont) phase results in clinical signs of systemic disease [1]. In *Cytauxzoon* sp. infection, the same development can be assumed, but the lack of histological examination in our cases did not allow evaluation of this data. In the cases reported here, laboratory abnormalities included non-regenerative anemia (cat n. 1 and n. 3), thrombocytopenia (cat n. 1 and n. 3), neutropenia (cat n. 3), leukopenia (cat n. 3), hyperbilirubinemia (cat n. 1), hyperglycemia (cat n. 2), hypoalbuminemia (cat n. 2) and, in all cases, mild elevations of hepatic enzyme activity and urea. 

These laboratory findings were consistent with previous reports. In particular, anemia is a recurrent finding, both in sick cats and in apparently healthy cats [46,48,49,51,55]. Normocytic, normochromic, non-regenerative anemia is a common finding during infection by *Cytauxzoon felis* and develops relatively late in the course of the disease [1]. This could, in part, be due to phagocytosis of red blood cells because erythro-phagocytosis is a prominent finding in many organs [1]. In our cases, it was not possible to associate non-regenerative anemia only with *Cytauxzoon* sp. infection because two anemic cats had co-infections (cat n. 1 with FIV and *Mycoplasma* sp. and cat n. 3 with FIV). Different feline hemoplasma species have different pathogenic features [73]. In particular, *Candidatus* Mycoplasma turicensis does not exclusively determine hemolytic anemia; the development of mild to moderate anemia could be due to predisposing concurrent disease or immune suppression [73,76,77,78,79]. Thrombocytopenia was present in the two anemic cats. Thrombocytopenia is seen in many cats with cytauxzoonosis. This may result from increased consumption of platelets due to DIC [1]. In our cases, no hemostatic profiles were performed to confirm this hypothesis, although the two thrombocytopenic cats showed no clinical signs related to DIC. Consistent with data reported in the literature, in our cases, hyperbilirubinemia, an increase in liver enzymes, hypoalbuminemia and hyperglycemia were nonspecific chemistry changes found [1]. The increased urea concentrations seen in all three cats might be due to dehydration, but renal azotemia could not be ruled out due to a lack of urinalysis. An increase in urea levels has been reported, but in only a few cases was this associated with an increase in creatinine concentrations that might be related to renal disease [46,55]. In the other cases, there was only a mild/moderate increase in azotemia of uncertain origin [48,49,55]. Molecular analyses, as expected, showed that isolates in our samples were different from *Cytauxzoon felis*. In the phylogenetic tree our sequences clustered in the group of Italian sequences of *Felis silvestris* belonging to the genotype EU1. This genotype is the most frequently found and is also present in Germany, Romania, the Czech Republic and Bosnia Herzegovina. However, the uniformity of the 18S rDNA sequences did not allow a clear genospecies delineation to separate the EU1 genotype from the EU2 minor genotype or the rare EU3 genotype, both present in Romania [38]. 

It is not yet known whether this new distinction between species is related to differential severity of the disease. This complicates the picture where there are co-infections with other pathogens.

In the present study, two cases (cat n. 1 and cat n. 3) were also positive for FIV infection and two cats (cat n. 1 and cat n. 2) were co-infected by *Candidatus* Mycoplasma turicensis.

In cytauxzoonosis, the role of coinfection is unclear. *Cytauxzoon* sp. seems less virulent than *Cytauxzoon felis* and it has been suggested that disease and clinical symptoms could develop preferentially in cases of concurrent disease or in cases of immunodeficiency [55]. Retroviral or haemoplasma co-infections could exacerbate clinical outcomes [51]; however, no previous relationship has been described between *Cytauxzoon* sp. and retroviral infections [55,80]. We assume that, as a result of the immunosuppression state due to FIV, the *Cytauxzoon* sp. could contribute as an opportunistic factor triggering the clinical pictures. However, the clinical role of *Cytauxzoon* sp. in domestic cats without immunosuppression and coinfections remains debatable [46]. 

To the best of our knowledge, in the domestic cat, no report of natural coinfection by *Cytauxzoon* sp. and *Candidatus* Mycoplasma turicensis has been described. There are only two Brazilian studies that have documented natural coinfection of *Cytauxzoon felis* and *Candidatus* Mycoplasma haemominutum in three domestic cats [81,82]. However, previous Italian studies showed that *Candidatus* Mycoplasma turicensis has a low prevalence, ranging from 0.2% to 5.1% [60,73,83,84].

All three cats received doxycycline as the first therapeutic regime. Regarding *Cytauxzoon* sp., different therapeutic treatments have been used with varying efficacy [48,49,50,55]. Recent studies have suggested that the most effective therapeutic treatment for acute *Cytauxzoon felis* infection is the combination of atovaquone and azithromycin [85]. Pending the results of the molecular investigation, the choice to use doxycycline was linked to the clinical conditions of the subjects, the suspicion of *Candidatus* Mycoplasma sp. infection, limited knowledge about therapy for *Cytauxzoon* sp. infection, and the limited economic resources of the owners. Unfortunately, we have no data about follow up and the efficacy of therapy cannot be reported.

The subjects reported in the present study were autochthonous cats. In Italy, studies have been conducted in different geographic areas with different results. A study on the endemicity of *Cytauxzoon* sp. in owned and colony domestic cats in the north-eastern area of Italy (Trieste) reported a 23% prevalence [55]. In contrast, in a recent study conducted in a larger area of north-eastern Italy (including Veneto, Friuli-Venezia Giulia, Trentino Alto-Adige regions) 3.8% positivity was found [61]. A positivity of 2.4% was found in another study, including other regions of northern Italy (Emilia Romagna region) [58]. Conversely, different epidemiological studies carried out in other areas of northern and central Italy have not detected the presence of *Cytauxzoon* sp. [57,59]. For example, in central Italy (Umbria region), in one study including 166 cats, no positivity was found for this pathogen [57], while another report describes two cases of *Cytauxzoon* sp. infection in free-ranging cats (Lazio region) [49].

This data supports the view that these hemoprotozoa occur in hyper-endemic foci or “hot spots” within the same country, as previously demonstrated in the USA [65]. This difference in prevalence between regions belonging to the same geographical area was accounted for by the presence of rural areas or cases of urban areas near wooded land, and to the lower use of pesticides in certain regions compared to cities [59]. 

In agreement with other reports, all cats described in the present study lived in rural areas with outdoor access. Furthermore, they received irregular preventative treatment against ectoparasites and did not have any travel history. At the time of admission, no cats showed the presence of ticks, but this means of transmission of the pathogen cannot be excluded as this lifestyle predisposes to higher exposure to tick vectors [37,55]. In particular, a significant association between the detection of *Cytauxzoon* sp. in domestic cats with an outdoor lifestyle has been reported, mostly in rural areas [55,56]. Although reservoirs in Europe have not been definitively identified, *Ixodes* spp. and/or *Dermacentor* spp. ticks could be involved in the transmission of *Cytauxzoon* sp. [75]. 

European domestic cats could be acting as reservoirs for *Cytauxzoon* sp. and could be a possible source of infection [37,55]. In Italy, the presence of *Cytauxzoon* sp. in some wild cats (*Felis silvestris silvestris*) was reported only in some regions (e.g., Marche, Latium, Friuli Venetia Giulia) [47]. In our cases, no contacts with wild animals were reported, but this route of transmission cannot be ruled out. Aggressive interactions could also be considered as a further means of transmission. This means of transmission has not been described for *Cytauxzoon* sp. but, interestingly, an experimental study showed that aggressive interactions (e.g., cat bites) can induce direct transmission of *Candidatus* Mycoplasma turicensis via exposure to blood from an infected cat [86]. This event could also be hypothesized for *Cytauxzoon* sp., as cats n. 1 and 2 were coinfected with *Candidatus* Mycoplasma turicensis. Although experimental studies failed to demonstrate perinatal transmission of *Cytauxzoon felis* in domestic cats [87], the transmission of *Cytauxzoon* sp. via vertical transmission, as well as via blood transfusion, has been documented [49,51]. However, in the present study, there was no previous data to support these hypotheses of transmission.

## 5. Conclusions

This report describes the accidental detection of *Cytauxzoon* sp. in domestic cats of central Italy without specific clinical signs. Furthermore, it provides the first report of coinfection with *Candidatus* Mycoplasma turicensis in two cats. 

Cytauxzoonosis should be included among differential diagnoses in nonendemic areas, such as Italy, in the presence of coinfections, in subjects with the possibility of contact with ticks, and in blood donors [88,89,90,91]. There is a significant need to improve knowledge of the distribution of vector-borne diseases affecting the autochthonous feline population and to describe the potential role of the cat as a reservoir in central Italy and the potential impact of the *Cytauxzoon* infection on the patient.

## Figures and Tables

**Table 1:** Cell blood count, biochemical analysis and rapid enzyme-linked immunosorbent assay test for FIV and FeLV of three domestic cats in central Italy.

Parameter	Cat n. 1	Cat n. 2	Cat n. 3	Reference Range
RBCs (×10^6^/mcL)	3.79	7.76	3.02	5.0–10.0
Hb (g/dL)	5.5	10.5	3.3	8.0–15.0
Hct (%)	17.4	31.9	10.4	24–55
MCV (fL)	45.9	41.1	34.4	39–55
MCH (pg)	12.9	14.5	13.5	13–18
MCHC (%)	31.6	32.9	31.7	30–36
RDW (%)	20.2	20.8	22.3	19.7–29.3
WBCs (×10^3^/mcL)	7.550	4.660	14.930	5.5–15.5
Neutrophils (n.)	4.832	2.970	2.514	2.500–12.800
Lymphocytes (n.)	1.963	1.560	12.386	1.500–7.000
Monocytes (n.)	604	350	0	0–850
Eosinophils (n.)	151	80	30	0–150
Basophils (n.)	0	0	0	0–100
PLTs (×10^3^/mcL)	12	508	49	300–800
MPV (fL)	22	17	15	11–18
AST (IU/L)	40	28	143	14–40
ALT (IU/L)	50	113	18	6–40
ALP (IU/L)	51	63	8	7–30
GGT (IU/L)		4	1	<10
Total bilirubin (mg/dL)	1.99	0.06	1.01	1.15–1.20
Cholesterol (mg/dL)	172	121	136	75–150
TGs (mg/dL)	64	24	53	50–100
Urea (mg/dL)	42	42	61	20–30
CPK (IU/L)	210	350	402	50–493
LDH (IU/L)	1480	234	3165	75–490
Creatinine (mg/dL)	1.09	1.18	0.71	1.0–2.0
Glucose (mg/dL)	100	132	51	64–120
Calcium (mg/dL)	8.9	10.6	7.0	7.0–10.0
Phosphorus (mg/dL)	4.6	5.6	4.8	2.5–5.0
Sodium (mEq/L)	150	152	139	138–152
Potassium (mEq/L)	4.2	3.8	3.8	3.4–5.1
Chloride (mEq/L)	110	118	115	100–120
TP (g/dL)	8.4	7.3	6.0	6.0–8.5
Albumin (g/dL)	2.7	3.3	1.4	2.1–3.3
Globulin (g/dL)	5.7	4.0	4.6	3.0–3.8
Albumin/Globulin	0.5	0.8	0.3	0.4–1.7
FIV	Positive	Negative	Positive	
FeLV	Negative	Negative	Negative	

RBCs, red blood cells; Hb, hemoglobin; Hct, hematocrit; MCV, mean corpuscular volume; MCH, mean corpuscular hemoglobin; MCHC, mean corpuscular hemoglobin concentration; RDW, RBC distribution width; WBC, white blood cells; PLTs, platelets; AST, aspartate transferase; ALT, alanine transferase; ALP, alkaline phosphatase; GGT, gamma-glutamyl transpeptidase; TGs, triglycerides; CPK, creatine phosphokinase; LDH, lactic dehydrogenase; TP, total protein; FIV; feline immunodeficiency virus; FeLV, feline leukaemia virus.

## Data Availability

All data are reported in the article.
